# Supplementary material for: Endosomal TLR3 co-receptor CLEC18A enhances host immune response to viral infection
Source: Commun Biol. 2021 Feb 18;4:229. doi: 10.1038/s42003-021-01745-7 (PMC7893028; doi:10.1038/s42003-021-01745-7)
Supplement: Supplementary file 1 — Supplementary Information [file 42003_2021_1745_MOESM1_ESM.pdf]

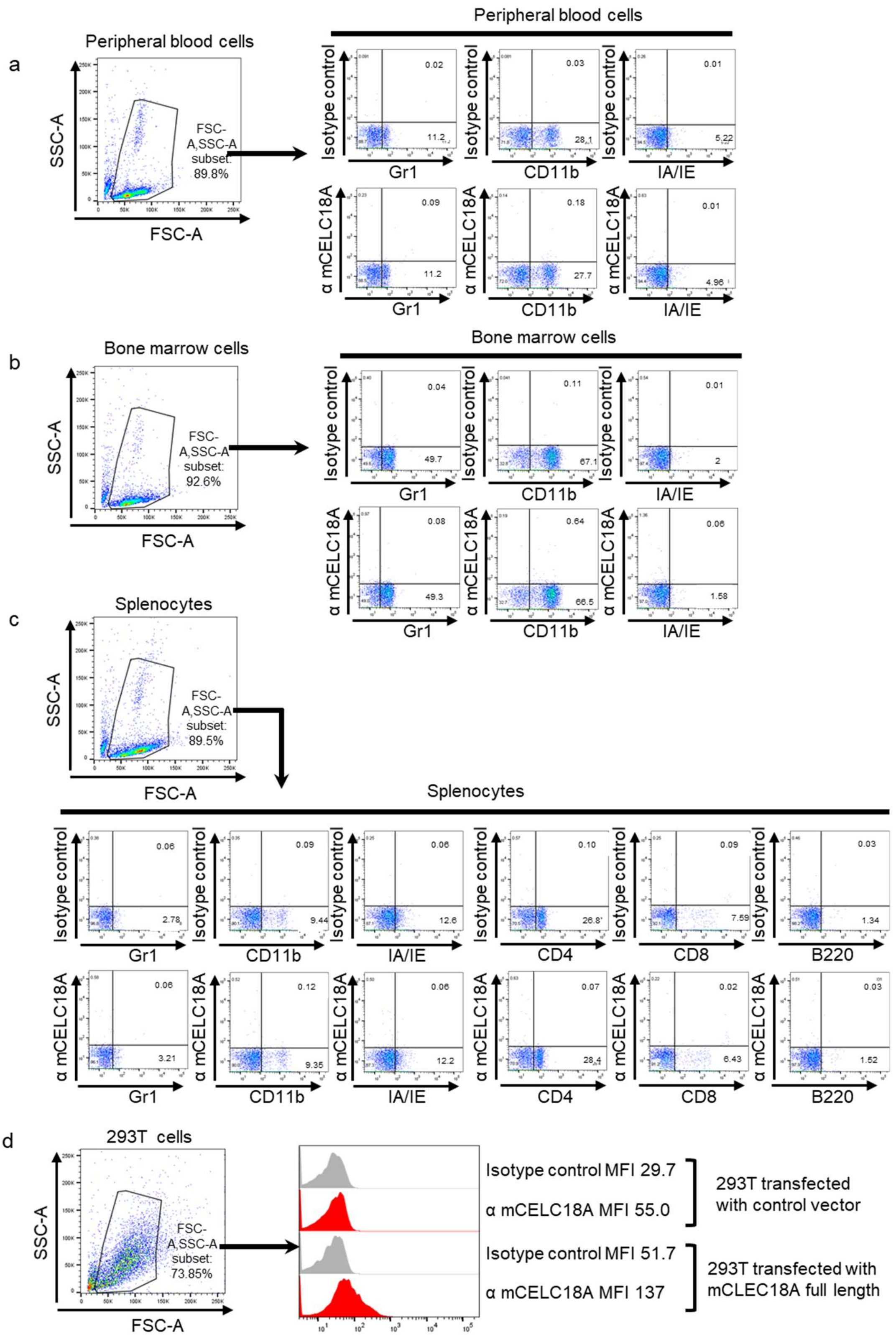

Supplementary Figure 1. Determination of mCLEC18A expression by flow cytometry

Mouse peripheral blood cells (a), bone marrow cells (b), and splenocytes (c) were

isolated from WT mice, followed by incubation with fluorochrome-conjugated mAb to detect various cell surface markers and endogenous intracellular CLEC18A by flow cytometry. (d) Detection of endogenous mCLEC18A by flow cytometry. The 293T cells were transfected with pCMV-Tag4A-mCLEC18A to express mCLEC18A as positive control for intracellular staining.

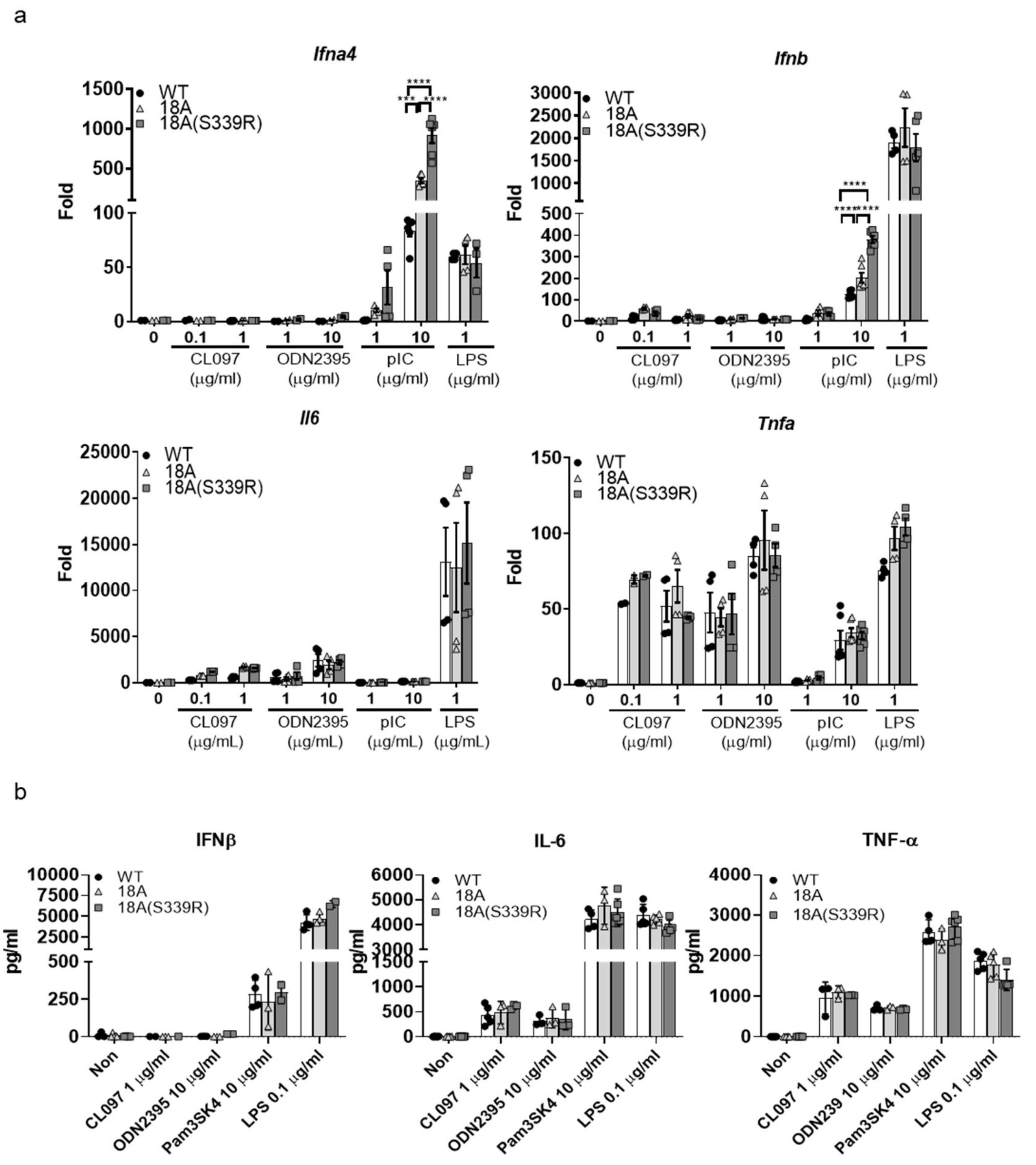

Supplementary Figure 2. Effect of TLR ligands to induce cytokine expression in BMDMs

BMDMs (1×10^6/well) from WT, ROSA-CLEC18A and ROSA-CLEC18A(S339R) mice were incubated with various TLR ligands for 12 or 24 hr, and samples were harvested to determine cytokine expression by RT-PCR (a) and ELISA (b) One-way ANOVA was performed. * p <0.05, **p<0.01, ***P<0.001, for WT group versus group of CLEC18A or CLEC18A(S339R). BMDMs: bone marrow derived macrophages.

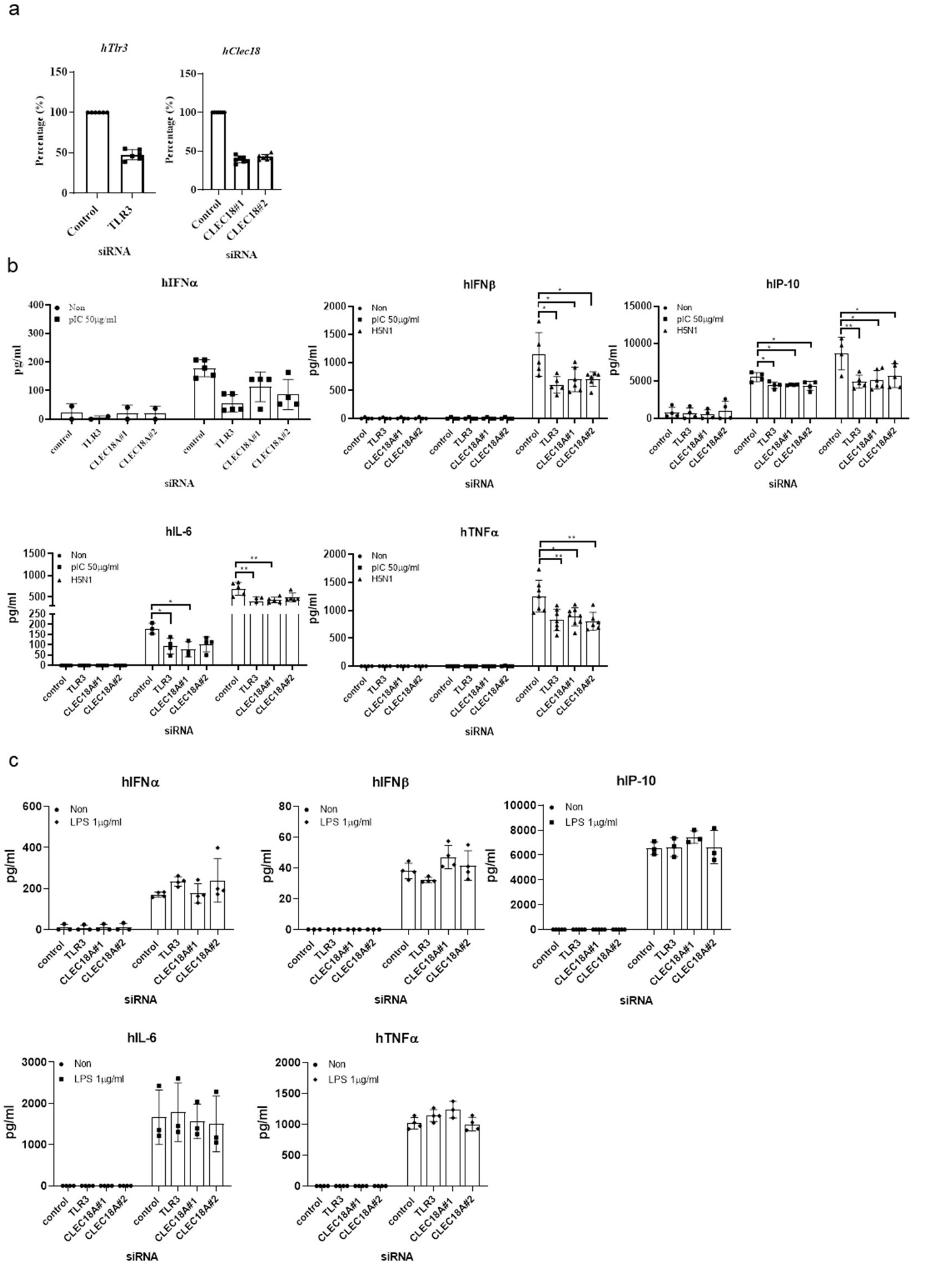

Supplementary Figure 3. CLEC18 enhances the expression of IFN and proinflammatory cytokines in human macrophages

(a) The expression level of *hTlr3* and *hCLEC18* in siRNA-transfected human macrophage cells. (b&c) Human macrophages (7×10^4/well) were transfected with human TLR3 or CLEC18 siRNA for 48 hr, followed by incubation with high

molecular weight poly (I:C) (50 μg/ml) and H5N1 virus (M.O.I. = 2) (b), or LPS (1 μg/ml) (c) for 24 hr. Samples were harvested to determine cytokine expression by ELISA. One-way ANOVA was performed. * $p < 0.05$, **$p < 0.01$, ***$P < 0.001$, for control group versus group of TLR3 or CLEC18 siRNA. pIC: poly (I:C).

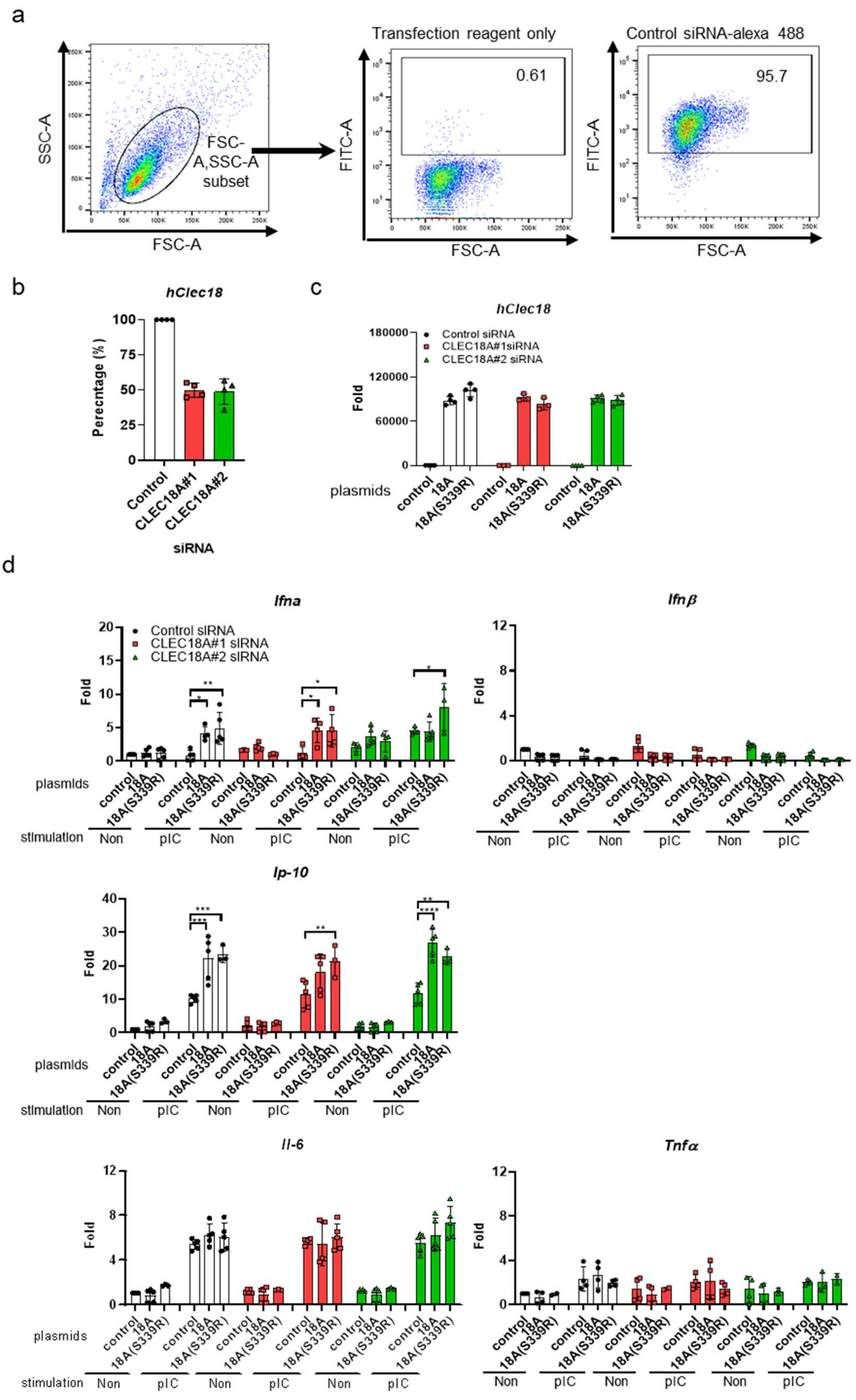

Supplementary Figure 4. CLEC18 enhances IFN cytokines production in HT1080 cell lines

(a) The transfection efficiency of control siRNA-alexa 488 is shown by flow cytometry. (b) The expression levels of *hCLEC18* in siRNA transfected HT1080 cells were determined by RT-PCR. (c) The expression level of *hCLEC18* in plasmid transfected cells that firstly transfect with siRNA was determined by RT-PCR. (d) The siRNA–transfected HT1080 cells were further transfected with control vector, pMACS.Kk-HA(C)-hCLEC18A and pMACS.Kk-HA(C)-hCLEC18A(S339R) for 24 h, and further incubated with high molecular weight poly (I:C) (50 μg/ml) for 8 h. Samples were harvested to determine cytokine expression by qPCR. Two-way ANOVA was performed. * $p <0.05$, **$p<0.01$, ***$P<0.001$, for control vectors group versus group of hCLEC18A or hCLEC18A(S339R) plasmids. pIC: poly (I:C). White color: HT1080 transfected with control siRNA; Red color: HT1080 transfected with CLEC18A#1 siRNA; Green color: HT1080 transfected with CLEC18A#2 siRNA;

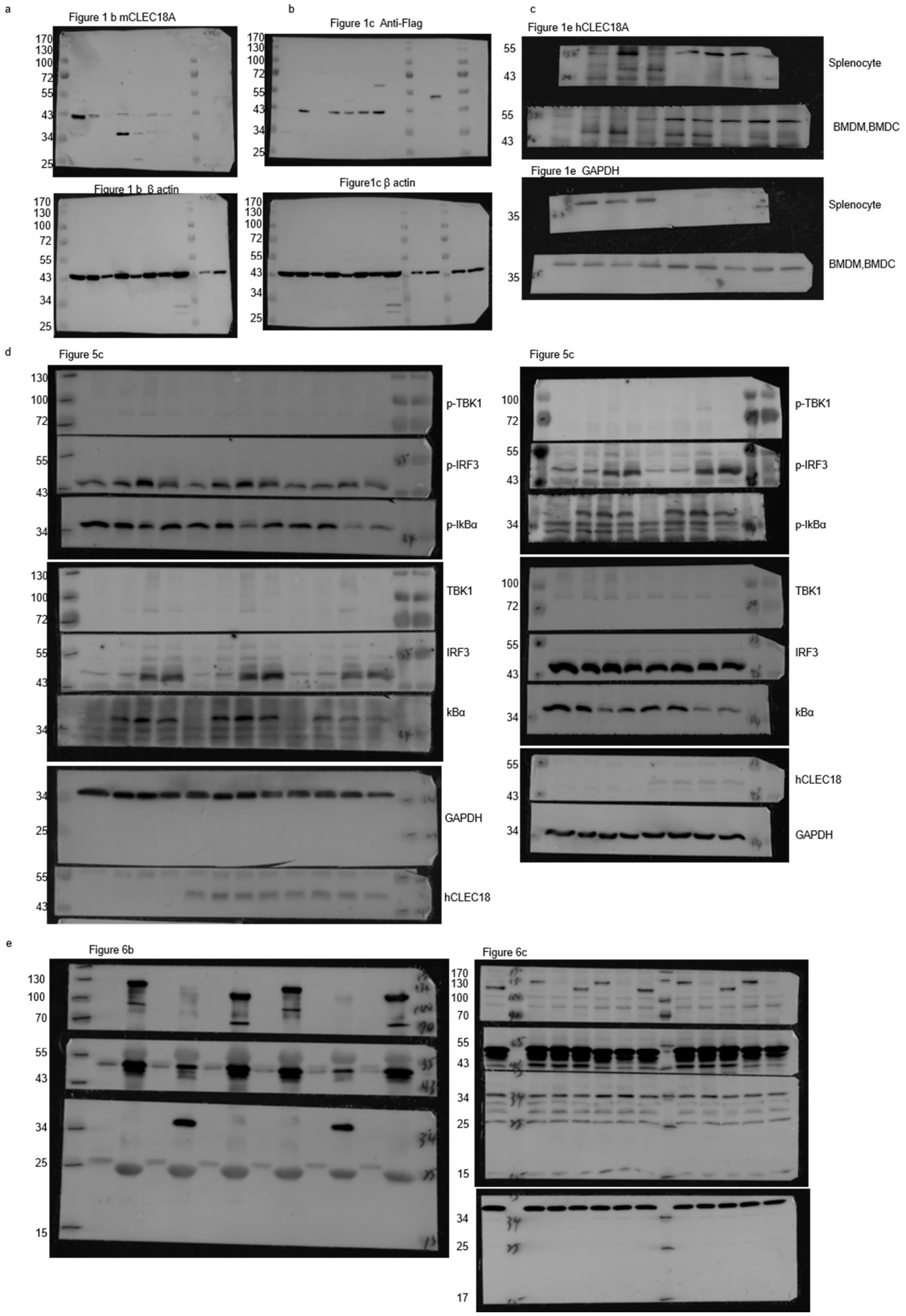
a
Figure 1 b mCLEC18A
170
130
100
72
55
43
34
25
Figure 1 b β actin
b
Figure 1c Anti-Flag
Figure1c β actin
c
Figure 1e hCLEC18A
Splenocyte
BMDM,BMDC
Figure 1e GAPDH
35
d
Figure 5c
p-TBK1
p-IRF3
p-IkBα
TBK1
IRF3
kBα
GAPDH
hCLEC18
e
Figure 6b
Figure 6c
15
17

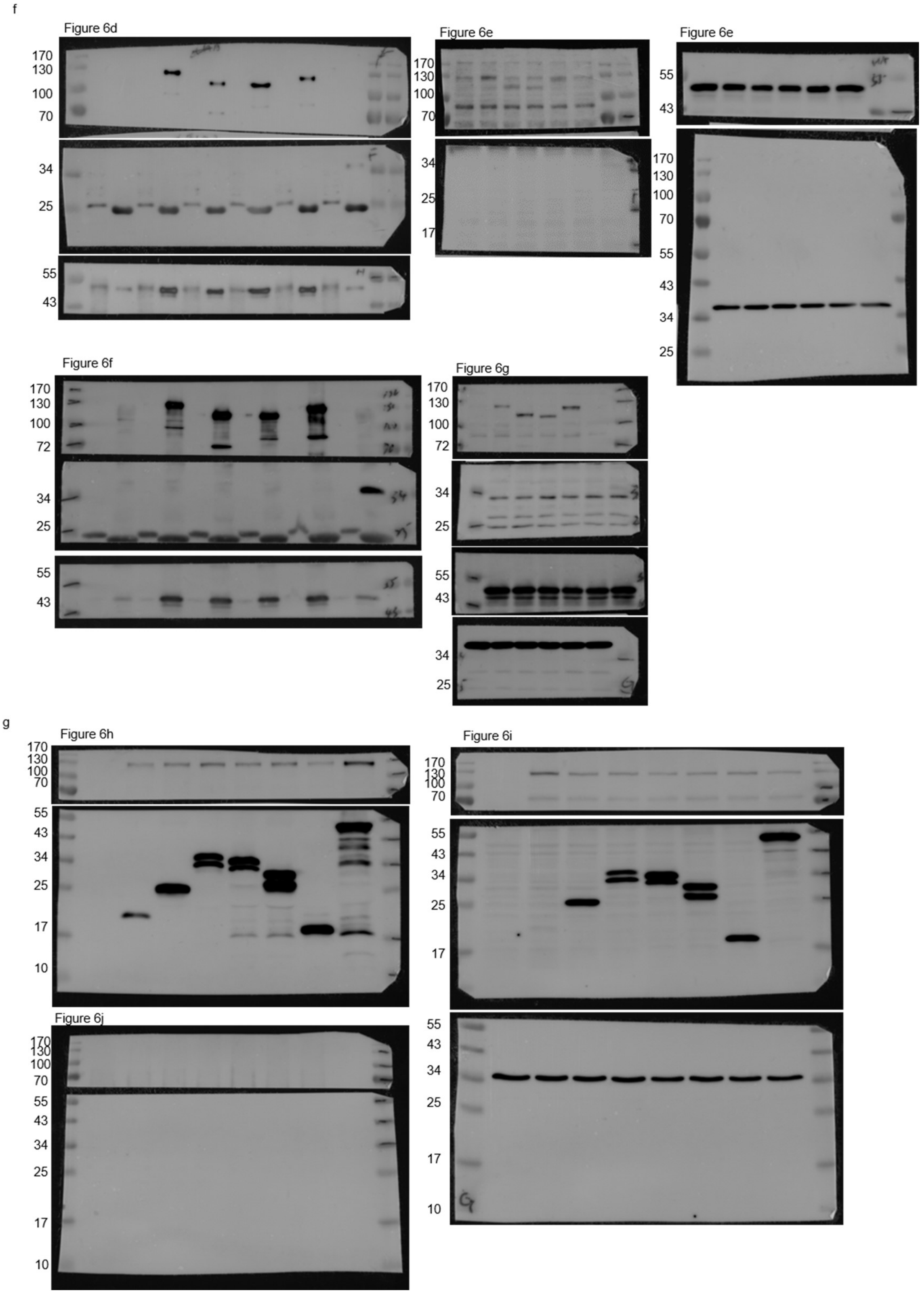

Supplementary Figure 5. Full blot shows in Fig1, Fig5, Fig6

(a~c) Blots shown in Figure 1b,c&e; (d) Blots shown in Figure 5c; (e~g) Blots shown in Figure 6 b~j.

Primer	Forward	Reverse
hGapdh	5’-ccactcctccacctttgac-3’	5’-accctgttgctgtagcca -3’
hIfnβ	5’-aaactcatgagcagtctgca -3’	5’- actatggtccaggcacagtg -3’
hIfna1/13	5’- tggctgtgaagaaatacttccg-3’	5’- tgttttcatgttggaccagatg-3’
hTnfα	5’ acaagcctgtagcccatgtt-3’	5’- aaagtagacctgcccagact-3’
hIl-6	5’-gtagccgccccacacaga-3’	5’-catgtctcctttctcagggctg -3’
mgapdh	5’-gcatccactggtgctgcc-3’,	5’-tcatcatacttggcaggtttc-3’
mIfnβ	5’-ccaccactcattctgaggca -3’	5’- atggtggtccgagcagagat-3’
mIfna4	5’-tactcagcagaccttgaacct-3’	5’-cagtcttggcagcaagttgac -3’
mIfnλ2/3	5`- agctgcaggccttcaaaaag -3`	5`- tgggagtgaatgtggctcag -3`
mTnfα	5’-gcctcttctcattcctgcttg -3’	5’-ctgatgagagggaggccatt -3’
mIl-6	5’-ctgcaagagacttccatccagtt-3’	5’-gaagtagggaaggccgtgg-3’
mMx1	5`-aaccctgctacctttcaa-3’	5`-aagcatcgttttctctatttc-3’
mCh25h	5’-tgctacaacggttcggagc -3’	5’-agaagcccacgtaagtgatgat -3’
mIsg15	5’-tgggacctaaaggtgaagatgctg-3’	5’-tgcttgatcactgtgcactggg -3’
M gene	5’-cttctaaccgaggtcgaaacg-3’	5’-ggcattttggacaaagcgtcta-3’
NP gene	5'-ttttctagcacggtctgcactcatattg-3'	5’-cttggctgttttgaagcagtctgaaag-3’

Supplementary Table I. Primer list

protein	Protein size (MW)	loading protein (ug)	Con.(nM)
ecdTLR3-Fc	130k	1.18	45.4
CLEC18A-Fc	55k	0.5	45.4
CLEC18A(S339R)-Fc	55k	0.5	45.4
dsRNA	dsRNA size (MW)	dsRNA strength (kb)	loading dsRNA (ug)
Poly(I:C) HMW	3135k	1.5-8 (ave=4.75kb)	10
pH 6.3 condition	kon(1/Ms)	kdis(1/s)	KD (M)
ecdTLR3-Fc	8.13E+05	4.54E-03	5.59E-09
CLEC18A-Fc	2.08E+06	4.37E-02	2.10E-08
CLEC18A(S339R)-Fc	2.38E+06	1.95E-02	8.17E-09
ecdTLR3-Fc+18A-Fc	5.89E+06	2.63E-03	4.46E-10
ecdTLR3-Fc+18A(S339R)-Fc	4.69E-03	<1.0E-07	<1.0E-12

Supplementary Table II. Determination of affinity of poly (I:C)-CLEC18 versus poly (I:C)-TLR3

Bio-layer interferometry (BLI, ForteBio) binding assays show the binding affinity between CLEC18-CTLD and ecdTLR3 fusion protein to HMW poly (I:C), respectively. The biotinylated-CLEC18A-CTLD-Fc, CLEC18A(S339R)-CTLD-Fc and ecdTLR3-Fc were immobilized on streptavidin (SA)-coated biosensor tips, respectively, followed by incubation with HMW poly (I:C) in PiBS binding buffer (20 μM Pipes, 150 mM NaCl, pH = 6.3). The processed data were fitted with the integrated fitting function by a 1:1 binding mode. The various KD values obtained by curve fitting were summarized in supplementary Table II. CTLD: C- type lectin like domain; ecd: extracellular domain; HMW: high molecular weight.
