# Supplementary material for: Endosomal TLR3 co-receptor CLEC18A enhances host immune response to viral infection
Source: Commun Biol. 2021 Feb 18;4:229. doi: 10.1038/s42003-021-01745-7 (PMC7893028; doi:10.1038/s42003-021-01745-7)
Supplement: Supplementary file 2 — Description of Additional Supplementary Files [file 42003_2021_1745_MOESM2_ESM.pdf]

Description of Additional Supplementary Files

File name: Supplementary Data 1

Description: The source data underlying graphs shown in main figures are presented in Supplementary Data 1.
