# Supplementary material for: Endosomal TLR3 co-receptor CLEC18A enhances host immune response to viral infection
Source: Commun Biol. 2021 Feb 18;4:229. doi: 10.1038/s42003-021-01745-7 (PMC7893028; doi:10.1038/s42003-021-01745-7)
Supplement: Supplementary file 4 — Reporting Summary [file 42003_2021_1745_MOESM4_ESM.pdf]

nature research

Corresponding author(s): Shie-Liang Hsieh

Last updated by author(s): Dec 22, 2020

Reporting Summary

Nature Research wishes to improve the reproducibility of the work that we publish. This form provides structure for consistency and transparency in reporting. For further information on Nature Research policies, see our Editorial Policies and the Editorial Policy Checklist.

Statistics

For all statistical analyses, confirm that the following items are present in the figure legend, table legend, main text, or Methods section.

n/a	Confirmed	
☐	☒	The exact sample size (*n*) for each experimental group/condition, given as a discrete number and unit of measurement
☐	☒	A statement on whether measurements were taken from distinct samples or whether the same sample was measured repeatedly
☐	☒	The statistical test(s) used AND whether they are one- or two-sided<br>*Only common tests should be described solely by name; describe more complex techniques in the Methods section.*
☐	☒	A description of all covariates tested
☐	☒	A description of any assumptions or corrections, such as tests of normality and adjustment for multiple comparisons
☐	☒	A full description of the statistical parameters including central tendency (e.g. means) or other basic estimates (e.g. regression coefficient) AND variation (e.g. standard deviation) or associated estimates of uncertainty (e.g. confidence intervals)
☐	☒	For null hypothesis testing, the test statistic (e.g. *F*, *t*, *r*) with confidence intervals, effect sizes, degrees of freedom and *P* value noted<br>*Give P values as exact values whenever suitable.*
☐	☒	For Bayesian analysis, information on the choice of priors and Markov chain Monte Carlo settings
☐	☒	For hierarchical and complex designs, identification of the appropriate level for tests and full reporting of outcomes
☐	☒	Estimates of effect sizes (e.g. Cohen's *d*, Pearson's *r*), indicating how they were calculated

Our web collection on statistics for biologists contains articles on many of the points above.

Software and code

Policy information about availability of computer code

Data collection	LightCycler480 software 1.5.1.62 (build-in software)<br>GraphPad Prism 8.2.1 GPS-1350867-LKQH-F0642<br>BD FACSuite v1.0.6 (build-in software)<br>FlowJo (AA040127000)
Data analysis	LightCycler480 software 1.5.1.62 (build-in software)<br>GraphPad Prism 8.2.1 GPS-1350867-LKQH-F0642<br>BD FACSuite v1.0.6 (build-in software)<br>FlowJo (AA040127000)

For manuscripts utilizing custom algorithms or software that are central to the research but not yet described in published literature, software must be made available to editors and reviewers. We strongly encourage code deposition in a community repository (e.g. GitHub). See the Nature Research guidelines for submitting code & software for further information.

Data

Policy information about availability of data

All manuscripts must include a data availability statement. This statement should provide the following information, where applicable:

- Accession codes, unique identifiers, or web links for publicly available datasets
- A list of figures that have associated raw data
- A description of any restrictions on data availability

The authors declare that the data supporting the findings of this study are available within the paper and its supplementary information files.

Field-specific reporting

Please select the one below that is the best fit for your research. If you are not sure, read the appropriate sections before making your selection.

- [x] Life sciences
- [ ] Behavioural & social sciences
- [ ] Ecological, evolutionary & environmental sciences

For a reference copy of the document with all sections, see nature.com/documents/nr-reporting-summary-flat.pdf

Life sciences study design

All studies must disclose on these points even when the disclosure is negative.

Sample size	The sample size is determined based on statistics significance of each experiment.
Data exclusions	No data are excluded
Replication	The data are repeated at least three times by the co-authors listed in the authorship
Randomization	Animal are chosen randomly based on the ages, not specific group.
Blinding	Nil

Reporting for specific materials, systems and methods

We require information from authors about some types of materials, experimental systems and methods used in many studies. Here, indicate whether each material, system or method listed is relevant to your study. If you are not sure if a list item applies to your research, read the appropriate section before selecting a response.

Materials & experimental systems

n/a	Involved in the study	
	x	Antibodies
	x	Eukaryotic cell lines
x		Palaeontology and archaeology
	x	Animals and other organisms
x		Human research participants
x		Clinical data
x		Dual use research of concern

Methods

n/a	Involved in the study	
x		ChIP-seq
	x	Flow cytometry
x		MRI-based neuroimaging

Antibodies

Antibodies used	Anti-phospho-TBK1 (#5483), anti-TBK1 (#3504), anti-phospho-IRF3 (#4947), anti-IRF3 (#4302), anti-phospho-IκBα (#9246), and anti-IκBα (#4814) Abs were purchased from Cell Signaling Technology. TLR3 antibody (GTX20260) and TLR7 antibody (NBP2-24906) purchased from Genetex and Novus Biologicals. PE-conjugated anti-mouse CD4 antibody (Biolegend 100408), PerCP-Cy5.5-conjugated rat anti-mouse CD8a (BD 561109), PE/Cyanine7-conjugated anti-mouse CD11b antibody (Biolegend 101216), Alexa Fluor 488-conjugated anti-mouse Ly6G/Ly6C (Gr-1) antibody (Biolegend 108417), brilliant Violet 510-conjugated anti-mouse IA/IE antibody (Biolegend 107636), brilliant Violet 421-conjugated anti-mouse/human CD45R/B220 antibody (Biolegend 103251) were purchased from biolegend and BD.
Validation	Phospho-TBK1/NAK (Ser172) (D52C2) XP® Rabbit mAb #5483<br>Application: Western Blotting (WB),Immunoprecipitation (IP), Immunofluorescence (IF),Flow Cytometry (F)<br>TBK1/NAK (D1B4) Rabbit mAb #3504 Application: WB,IP<br>Phospho-IRF-3 (Ser396) (4D4G) Rabbit mAb #4947 Application:WB<br>IRF-3 (D83B9) Rabbit mAb #4302 Application: WB,IP<br>Phospho-IκBα (Ser32/36) (5A5) Mouse mAb #9246 Applicaiton:WB<br>IκBα (L35A5) Mouse mAb (Amino-terminal Antigen) #4814 Application:WB,IHC,IP,IF,F<br>TLR3 antibody (GTX20260) Rabbit mAb Application: WB,ICC/IF,IHC,ELISA<br>TLR7 antibody (NBP2-24906) Rabbit polyclone Ab Application: WB, DB, Flow, Flow-IC, ICC/IF, IHC, IHC-Fr, IHC-P, IP, PLA<br>PE-conjugated anti-mouse CD4 antibody (Biolegend 100408) Applicaiton:FC<br>PerCP-Cy5.5-conjugated rat anti-mouse CD8a (BD 561109) Applicaiton:FC<br>PE/Cyanine7-conjugated anti-mouse CD11b antibody (Biolegend 101216) Applicaiton:FC<br>Alexa Fluor 488-conjugated anti-mouse Ly6G/Ly6C (Gr-1) antibody (Biolegend 108417) Applicaiton:FC<br>brilliant Violet 510-conjugated anti-mouse IA/IE antibody (Biolegend 107636) Applicaiton:FC<br>brilliant Violet 421-conjugated anti-mouse/human CD45R/B220 antibody (Biolegend 103251) Applicaiton:FC

Eukaryotic cell lines

Policy information about cell lines

Cell line source(s)	ATCC
Authentication	The cell lines are from ATCC.
Mycoplasma contamination	The cell lines are free of mycoplasma contamnination. N.A.
Commonly misidentified lines (See ICLAC register)	N.A.

Animals and other organisms

Policy information about studies involving animals; ARRIVE guidelines recommended for reporting animal research

Laboratory animals	Eight to twelve-week-old wild-type C57BL/6
Wild animals	N.A.
Field-collected samples	N.A.
Ethics oversight	Animal experiments were approved by the Institutional Animal Care and Use Committee (IACUC) at AS core (protocol ID 18-12-1243), and in accordance with the recommendations in the Guide for the Care and Use of Laboratory Animals of the Taiwanese Council of Agriculture.

Note that full information on the approval of the study protocol must also be provided in the manuscript.

Flow Cytometry

Plots

Confirm that:

- [x] The axis labels state the marker and fluorochrome used (e.g. CD4-FITC).
- [x] The axis scales are clearly visible. Include numbers along axes only for bottom left plot of group (a 'group' is an analysis of identical markers).
- [x] All plots are contour plots with outliers or pseudocolor plots.
- [x] A numerical value for number of cells or percentage (with statistics) is provided.

Methodology

Sample preparation	Mouse peripheral blood cells, bone marrow cells, and splenocytes were isolated from WT mice, after RBC lysis, followed by incubation with fluorochrome-conjugated mAb to detect various cell surface markers and endogenous intracellular CLEC18A by flow cytometry.
Instrument	Genomics Research Center, Academia Sinica, Taipei, Taiwan
Software	BD FACSuite v1.0.6; FlowJo_V10 6.2
Cell population abundance	Mouse peripheral blood cells, bone marrow cells, and splenocytes were isolated without sorting.
Gating strategy	In this sample gating, cells were gated for total cells (FSC-A vs. SSC-A).

- [x] Tick this box to confirm that a figure exemplifying the gating strategy is provided in the Supplementary Information.
